# Changes in Saliva Analytes in Pigs in Different Clinical Situations from Farms Positive to Porcine Reproductive and Respiratory Syndrome (PRRS): A Pilot Study

**DOI:** 10.3390/v17060833

**Published:** 2025-06-09

**Authors:** Eva Llamas-Amor, Silvia Martínez-Subiela, Fernando Tecles, Aida Miralles, Elena Goyena, Andrea Martínez-Martínez, José Joaquín Cerón, Alberto Muñoz-Prieto

**Affiliations:** 1 Interdisciplinary Laboratory of Clinical Analysis (Interlab-UMU), Regional Campus of International Excellence ‘Campus Mare Nostrum’, University of Murcia, 30100 Murcia, Spain; eva.llamasa@um.es (E.L.-A.); silviams@um.es (S.M.-S.); ftecles@um.es (F.T.); jjceron@um.es (J.J.C.); 2 Department of Anatomy and Comparative Pathological Anatomy, Faculty of Veterinary Medicine, University of Murcia, 30100 Murcia, Spain; aida.miralles@um.es (A.M.); goyena@um.es (E.G.); 3 Agropecuaria Casas Nuevas Ltd., 30320 Murcia, Spain; amartinez@grupo-frances.es

**Keywords:** saliva, biomarkers, PRRSV, pig

## Abstract

Porcine reproductive and respiratory syndrome (PRRS) is aworldwide spread disease. This study analyzed the changes in saliva analytes of pigs infected with PRRS virus (PRRSV) in different clinical conditions that can appear in PRRSV-positive farms. Biomarkers for inflammation (haptoglobin, total proteins), immune response (adenosine deaminase), tissue damage (lactate dehydrogenase), stress (alpha-amylase), and sepsis (calprotectin, aldolase, Serpin B12) were measured in pigs under three clinical scenarios: (1) no evident clinical signs, (2) clinical signs indicating PRRSV activation, and (3) secondary bacterial infection by *Streptococcus suis*. Haptoglobin and lactate dehydrogenase showed significant increases in pigs with PRRSV activation compared to pigs without clinical signs. Additionally, the levels of Serpin B12, aldolase, calprotectin, total proteins, and the activity of adenosine deaminase significantly increased in pigs with meningitis compared to pigs without clinical signs, but did not show significant differences between healthy pigs and those with PRRSV clinical signs without bacterial infection. In summary, PRRSV-infected pigs can show differences in selected saliva analytes depending on their clinical condition. These findings may have practical applications for detecting PRRSV infections and differentiating cases with associated meningitis.

## 1. Introduction

Porcine reproductive and respiratory syndrome (PRRS) is a disease widely distributed in pig farms [1]. It is currently endemic in many countries and is one of the most important pathologies in the nursery and fattening phase, being a great threat to the swine industry worldwide [2,3], causing severe economic losses due to the worse performance of pigs and failure in the treatments of bacterial coinfection [3,4]. This syndrome is caused by an Arterivirus of single-stranded RNA that targets the host’s immune cells, producing apoptosis and alterations in immune modulation [5], leading to an increase in susceptibility to other pathogens that are present in farms [6].

In a PRRSV-positive farm, pigs can be in three different situations: (a) without overt clinical signs [7]; (b) with overt clinical manifestations of the disease, including respiratory lesions such as interstitial pneumonia that results in growth retardation and increased mortality in pigs of all ages, especially piglets [8]; and (c) with PRRSV and secondary infections such as *Streptococcus suis* (*S. suis*), influenza A virus, or atypical porcine pestivirus [9,10].

Saliva sampling is a non-invasive, easy, cheap, and fast method that does not require special training [11,12]. The use of saliva as a source of biomarkers for animal health has been increasing in the last few years, especially in pigs, where blood collection is highly stressful [12]. In this species, different biomarkers of inflammation, the immune system, tissue damage, stress, or sepsis can be measured in saliva. Examples of these biomarkers for inflammation are haptoglobin (Hp) and total proteins; for the immune system, one example is adenosine deaminase (ADA); for tissue damage, lactate dehydrogenase (LDH); for stress, alpha-amylase (sAA); and for sepsis, examples are calprotectin (S100A8-A9, Calp), aldolase, and Serpin B12. These biomarkers have been measured in different naturally occurring diseases, such as meningitis by *S. suis* [13], diarrhea by *Escherichia coli* (*E. coli*) [14], and in an experimental model of sepsis [15]. In PRRSV, the concentracions of acute-phase proteins such as Hp and C-reactive protein (CRP) [16] have been measured in the saliva of pigs with this disease; however, to the authors’ knowledge, there are no studies about the changes in other analytes that can be measured in saliva and no studies about the variation in salivary analytes in different clinical pictures that can appear in this disease.

This study aimed to investigate the changes that can occur in analytes in saliva across different clinical presentations observed in PRRSV-positive farms. For this purpose, a profile integrating Hp, total proteins, ADA, LDH, sAA, Calp, aldolase, and Serpin B12 was measured in pigs with PRRS in three different situations: (1) with no evident clinical signs of disease, (2) with clinical signs associated with PRRSV, and (3) with PRRSV and clinical signs of an associated *S. suis* coinfection.

## 2. Materials and Methods

### 2.1. Animals

This study was carried out with 68- to 78-day-old pigs (Landrace–Large White × Duroc) from two commercial nursery farms in southeastern Spain, which were positive for PRRSV with recirculations appearing at mid-transition. In both farms, pigs were housed in flatbed pens in groups of 19 piglets per pen and had ad libitum access to food (‘Starter’ with 17.4% crude protein, 4.866% crude fat, 3.5% crude fiber, and 1460 Kcal of metabolizable energy) and water. The pens were 2.3 × 2.6 m and in separate rooms with two corridors, and there were twenty-four pens in each room. The environmental control included two types of heating (underfloor heating in the center of each pen and space heating by delta tubes in the whole room), as well as cooling for the summer months using misters in the pens and in the central corridors to keep a continuous temperature of 25–26 °C. Ventilation was forced, and temperature control was automated by air heaters.

In both farms, the piglets were weaned at 28 days of age and transported together in trucks to a nursery unit, where they were housed and commingled in different pens without considering the litter effect. In addition, both farms did not vaccinate against PRRSV, and the piglets received the vaccines for *Circovirus* and *Mycoplasma hyopneumoniae* at weaning at 28 days of age.

The capacity of the first farm was 3000 piglets, and the second farm was 4320. Three different groups were formed as follows:

- Group A, without overt clinical signs of PRRSV (n = 16), included pigs that did not show any external clinical signs at physical examination. These pigs showed positive results for PRRS based on an RT-PCR of saliva (median = 34.30; IQR = 27.10–38.30).
- Group B, with clinical signs compatible with a PRRSV infection (n = 29), included pigs with clinical signs such as anorexia, growth retardation, dyspnea, and/or hirsutism and positive to PRRSV based on an RT-PCR of saliva (mean = 31; IQR = 26.8–33).
- Group C, with clinical signs compatible with PRRSV and *S. suis* infection (n = 20), consisting of fever, nervous signs compatible with meningitis, and/or arthritis. These pigs were classified by a previously reported severity scale that showed three pigs in grade 2, four pigs in grade 3, eight pigs in grade 4, and five pigs in grade 5 [13]. In four pigs of this group that died in this outbreak of this disease, the lungs were positive for *S. suis* and PRRSV by RT-PCR (mean *S. suis* Cts = 30.76 and PRRSV Cts = 23.34). These pigs were positive for PRRSV based on a TR-PCR of saliva (mean = 30.90; IQR = 26–37).

Groups A and B were located at one farm, and group C was located at another farm where an *S. suis* outbreak occurred.

All procedures were approved by the Ethical Committee on Animal Experimentation (CEEA) of the University of Murcia (protocol code CEEA 563/2019).

### 2.2. Biological Sampling and Pathogen Detection Analysis

Blood samples were taken from the three groups of animals in Vacuette^®^ tubes (Greiner Bio One España, SAU, Madrid, Spain) without additives for the extraction of venous blood by a vacuum system. In blood, a profile of pathogens including porcine influenza A virus, *Glasserella parasuis*, *Mycoplasma hyorhinis*, or PRRSV American was tested and showed negative results on RT-PCR. In the case of group C, blood samples were cultured on Columbia blood agar plates (Oxoid Ltd., Madrid, Spain) supplemented with 5% defibrinated pig blood for 48 h at 37 °C under aerobic conditions. The identification of isolates was carried out using standard methods and confirmed by PCR targeting the glutamate dehydrogenase gene, as previously outlined. All the pigs in the meningitis group tested positive for *S. suis* serotype 9.

Saliva was collected from the pigs using a metal rod fitted with a sponge, which was gently inserted into their mouths. After collection, the sponges were transferred into Salivette tubes (Sarstedt, Aktiengesellschaft & Co., Nümbrecht, Germany). The samples were immediately placed in a portable cooler maintained at 4–8 °C and kept there during transport to the laboratory. Upon arrival, the tubes were centrifuged at 3000× *g* for 10 min at 4 °C to extract the saliva supernatant. The resulting fluid was then aliquoted into Eppendorf tubes and stored at −80 °C for later analysis.

Saliva samples were analyzed by real-time PCR for the detection of PRRS viral particles with the commercial kit VetMAX™ PRRSV EU & NA 3.0 (Thermo Fischer Life Technology, Madrid, Spain) following the manufacturer’s instructions.

RNA extraction was performed using 250 μL of saliva and 750 μL of TRIzol LS reagent (Thermo Fischer Life Technology) following the manufacturer’s instructions and resuspending the RNA pellet in 40 μL of nucleic acid-free MilliQ water.

The PRRSV species was confirmed by RT-PCR as the European species in both cases and was negative for the American species.

All saliva samples were collected at the same time of day (9:00 a.m.) following previous recommendations in order to not include a possible bias due to the fluctuations in the analytes in saliva that can occur during different times of the day [17], and the sick pig group samples were collected before any treatment was administered.

The lung samples of the four deceased animals were analyzed by real-time PCR for the detection of PRRS viral particles (Thermo Fischer Life Technology) and *Streptococcus suis* (Zootecnia laboratory S.L.P., Salamanca, Spain), considering positive samples with a Ct value less than or equal to 33.

### 2.3. Biochemical Analysis of Saliva

#### 2.3.1. Biomarkers of Inflammation

Haptoglobin was measured by an immunological assay based on AlphaLisa technology previously used in saliva [18].

Total proteins were analyzed using a spectrophotometric assay to measure Low-Complexity Region (LCR) proteins (protein in urine and CSF, Spinreact, Girona, Spain), which had been previously validated in porcine saliva [13].

#### 2.3.2. Biomarkers of Immune System

Adenosine deaminase was analyzed with a commercially available spectrophotometric automated assay (Adenosine Deaminase assay kit, Diazyme Laboratories, Poway, CA, USA), previously validated in porcine saliva [19].

#### 2.3.3. Biomarkers of Tissue Damage

Lactate dehydrogenase was measured by a spectrophotometric assay using a commercial kit from Biosystem (Biosystem S.A., Barcelona, Spain) [20].

#### 2.3.4. Biomarkers of Stress

Alpha-amylase activity was measured by a commercial spectrophotometric assay (a-Amylase, OSR6182, Beckman Coulter, Singapore), previously validated in porcine saliva [21].

#### 2.3.5. Biomarkers of Sepsis

Calprotectin was analyzed using the BÜHLMANN fCal Turbo^®^ assay kit (BÜHLMANN, Laboratories AG, Schönenbuch, Switzerland), which is an immunoturbidimetric assay previously validated in porcine saliva [14].

Aldolase was measured by a spectrophotometric assay through a commercially available reagent kit (Aldolase, Randox Laboratories Ltd., Crumlin, UK) [22].

Serpin B12 was analyzed using an in-house immunological method based on amplified luminescent proximity homogeneous technology (AlphaLisa) [23].

All methods were performed using an Olympus AU400 autoanalyzer, except for those for Hp and Serpin B12.

#### 2.3.6. Statistical Analysis

Data were assessed for normality by the Shapiro–Wilk method and showed a non-normal distribution. A non-parametric approach was followed to analyze all the results. A Kruskal–Wallis test, following Dunn’s multiple comparisons with Bonferroni correction, was used to compare the differences in each variable between groups. The results were expressed as the median and interquartile range (IQR). The significance level was set at α = 0.05. In order to control Type II error, the effect size (r) was calculated for each pairwise comparison [24]. The value of r was interpreted as follows: <±0.3 as a low effect, between ±0.3 and ±0.5 as a medium effect, and >±0.5 as a large effect. All the statistical calculations were made using commercially available software (GraphPad Prism 9, GraphPad Software, San Diego, CA, USA; IBM SPSS Statistics for Windows, Version 26.0. IBM Corp., New York, NY, USA).

A sample size of 66 per group was calculated in order to achieve 80% statistical power. These calculations were performed using the TTestIndPower function from the statsmodels Python package (Python 3.10 version).

## 3. Results

**Figure 1:**
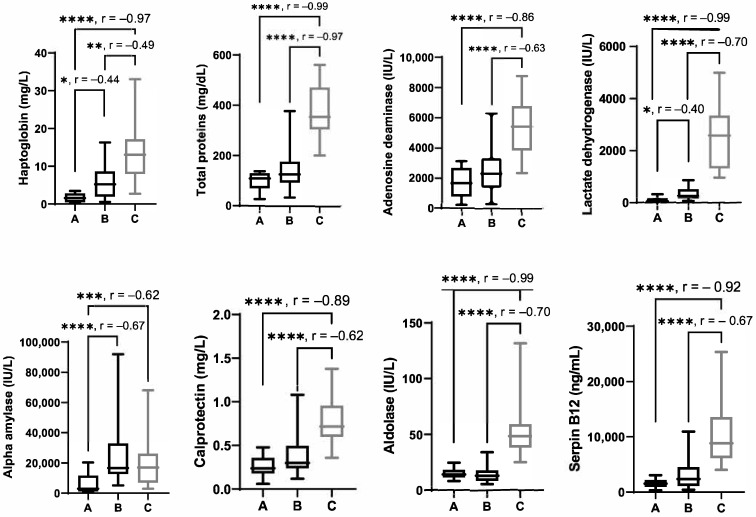
Analyte concentrations in the saliva of (A) pigs with no signs, (B) pigs infected by porcine respiratory and reproductive syndrome (PRRS) virus with clinical signs, and (C) pigs with coinfection by PRRSV and *S. suis*. Horizontal lines represent the median concentrations, boxes indicate the 25–75 percentiles, and whiskers represent the ranges; * *p* < 0.05, ** *p* < 0.01,*** *p* < 0.001, and **** *p* < 0.0001. r is the size effect.

The results of the biomarkers appear in Figure 1.

Salivary Hp concentrations were significantly higher in pigs with *S. suis* (median = 13.04 mg/L; IQR = 7.95–17.16) compared with pigs with clinical signs of PRRSV (median = 5.20 mg/L; IQR = 1.99–8.66) (*p* = 0.0021) and compared with non-sick pigs (median = 1.61 mg/L; IQR = 0.48–2.85) (*p* ≤ 0.0001). In addition, Hp levels were higher in pigs with clinical signs of PRRSV than in non-sick pigs (*p* = 0.01).

Salivary total protein concentrations were significantly higher in pigs with *S. suis* (median = 353.6 mg/dL; IQR = 305–470.9) when compared with pigs with clinical signs of PRRSV (median = 125.1 mg/dL; IQR = 92.87–377.2) (*p* < 0.0001) and compared with non-sick pigs (median = 109.9 mg/dL; IQR = 70.20–129.5) (*p* < 0.0001) but did not show differences between PRRSV and non-sick pigs.

Salivary ADA concentrations were significantly higher in pigs with *S. suis* (median = 5421 IU/L; IQR = 3846–6778) compared to pigs with clinical signs of PRRSV (median = 2307 U/L; IQR = 1395–3333) and non-sick pigs (median = 1660 IU/L; IQR = 787.7–2681) (*p* < 0.0001). There were no significant differences between pigs with clinical signs of PRRSV and non-sick pigs.

LDH activity was higher in the group with *S. suis* (median = 2583 IU/L; IQR = 1320–3347) than in pigs with clinical signs of PRRSV and non-sick pigs (*p* < 0.0001 in both cases). Salivary LDH concentrations were significantly higher in pigs with clinical signs of PRRSV (median = 258.9 IU/L; IQR = 168.3–517.9) compared with non-sick pigs (median = 54.90 IU/L; IQR = 36.8–185) (*p* = 0.0292).

sAA in pigs with *S. suis* (median = 16,982 IU/L; IQR 6912–26,106) showed higher values than non-sick pigs (*p* = 0.0009), but no differences with respect to the group with clinical signs of PRRSV were detected. sAA concentrations were significantly higher in pigs with PRRSV (median = 16,515 IU/L; IQR = 12,625–32,963) compared with non-sick pigs (median = 2870 IU/L; IQR =1142–11,507) (*p* < 0.0001).

In the biomarkers of sepsis, pigs with *S. suis* (median = 0.72 mg/L; IQR = 0.6–0.96) showed significantly higher values of Calp concentrations compared with the other two groups (*p* < 0.0001 in both cases). Salivary Calp concentrations did not show significant differences (*p* = 0.0503) between pigs with PRRSV (median = 0.3 mg/L; IQR = 0.24–0.495) and non-sick pigs (median = 0.24 mg/L; IQR = 0.18–0.36). Pigs with meningitis showed significantly higher values of aldolase than the other two groups (median = 48.40 IU/L; IQR = 38–59.10) (*p* < 0.0001 in both cases). Salivary aldolase concentrations did not show differences in pigs with PRRSV (median = 12.90 IU/L; IQR = 8.475–17.78) compared with non-sick pigs (median = 14.10 U/L; IQR = 11.30–18.20) (*p* = 0.99). In the *S. suis* group), Serpin B12 showed significantly higher values (median = 8838 ng/mL; IQR = 6173–13,590) than those of the other two groups (*p* < 0.0001 in both cases). It did not show significant changes in pigs with PRRSV (median = 2360 ng/mL; IQR = 1119–4533) compared with non-sick pigs (median = 1504 ng/mL; IQR = 948.8–2155).

All significant differences in the analytes of this study had an r value of >±0.3, indicating a moderate to high size effect.

## 4. Discussion

In this report, the changes occurring in saliva markers related to inflammation, immunity, stress, tissue damage, and sepsis in pigs with different clinical conditions in PRRS-positive farms were evaluated. PRRSV was evaluated by RT-PCR in all the pigs of this study in saliva. Previous reports have indicated that PRRSV can be quantified in saliva samples, which can be an alternative to serum [25,26].

Three groups of analytes could be made based on the changes shown in the pigs of this study:

One group included analytes that showed significant increases in the two groups of pigs with clinical signs (the group with clinical signs of PRRSV and also in the group of pigs with meningitis due to *S. suis* infection) compared to the non-sick pigs but that also showed increases of a higher magnitude in the pigs with meningitis. These analytes were Hp and LDH.

Regarding Hp, the pigs that did not show clinical signs in our study had Hp concentrations in saliva in the range of those of healthy pigs of the same age [27]. Based on the fact that Hp is an acute-phase protein whose levels increase in inflammatory conditions, the lack of an increase in Hp in our report indicated that the pigs without clinical signs did not have inflammation. Also, LDH, which is a marker related to cell injury [28], showed values in the range of those reported in healthy pigs [22], indicating the lack of evident tissue damage. However, the levels of these two biomarkers were significantly higher in the pigs with PRRSV showing clinical signs of PRRSV and also in pigs with meningitis due to *S. suis* infection. This agrees with previous reports in which Hp was increased in the serum of pigs with PRRSV and clinical signs [29,30]. In addition, it was demonstrated in another study that Hp was more sensitive than other acute-phase proteins, such as C-reactive protein (CRP) or serum amyloid A, in cases of PRRSV infection, and it could discriminate between sick and non-sick pigs, being correlated with the RT-PCR values in serum [31]. Also, LDH increases in pigs with *S. suis* and other infectious diseases such as *E. coli* [13,27,32]. The increases seen in Hp and LDH indicate the presence of inflammation and tissue damage in pigs with PRRSV and pigs with meningitis that could be a consequence of the “cytokine storm” produced in these diseases [33], involving various cytokines, such as IL-6, IL8, and TGF-B [34,35], being this “storm” in the case of meningitis of a higher magnitude.

The second group included analytes that showed significant increases in the group of pigs with meningitis compared to non-sick pigs and pigs with clinical signs of PRRS. These analytes were aldolase, Calp, Serpin B12, total protein, and ADA. One reason for these increases could be, in the case of aldolase, Calp, and Serpin B12, the fact that they are considered specific biomarkers of bacterial infection, which is a process involved in meningitis by *S. suis*. Aldolase is a marker of sepsis [36], whose levels have been reported to increase in bacterial infection in pigs [14]. Calp, also named S100/A8/A9, despite its expression can be stimulated in macrophages infected by PRRSV [37], is a protein whose levels did not show increases in viral diseases compared to bacterial diseases [38]. In addition, Serpin B12 concentrations have been demonstrated to increase in pigs with experimentally induced sepsis [23]. The lack of increase in aldolase and Serpin B12 and total protein in the saliva of pigs with PRRS should be further explored, and the mechanism for this elucidated. In addition, further validation is needed to establish the true specificity of these analytes for bacterial coinfections in PRRSV-positive pigs.

On the other hand, the biological mechanism of the changes in ADA can be different. The levels of this enzyme are higher in lymphoid organs, and this enzyme has a role in immune activation and has been proposed as an inflammatory analyte in pigs [39]. However, on the other hand, lower levels of ADA are associated with immune deficiency in humans [40]. The values of ADA in the pigs with clinical signs of PRRSV in our report are similar to those of the pigs without clinical signs and to those of healthy pigs reported in other studies [14]. This lack of significant changes in ADA in the pigs with clinical signs of PRRSV could indicate a possible balance between the decrease that could be produced by the destruction of lymphocytes by PRRSV [41], since it generates a decrease in lymphocyte numbers, that would lead to a decrease in this enzyme [42], and the increase that should occur due to the inflammation occurring in these diseased pigs indicated by the increase in Hp [43]. Therefore, the lymphoid depletion caused by the virus could be the cause of the lack of increase in ADA levels, which usually increase when there is inflammation [44]. In this line, in pigs with meningitis, there is an increase in ADA, probably due to the stimulation of T lymphocytes, as has been previously reported in pigs [10]. The inhibition of the immune system could also influence the lack of increases in total proteins found in our report in pigs with PRRSV, since the values of total proteins usually increase in inflammatory and infectious diseases [13], as occurred in the pigs with *S. suis* in our report.

sAA was the only analyte that had increases of a similar magnitude in pigs with PRRSV and pigs with *S. suis* infection. Increases in this enzyme have been related to stress associated with pain in pigs [45]. The higher value found in our report might be due to the stress and discomfort that produced clinical signs in the animals [46]. The mechanism that could be postulated to be responsible for the increases in sAA in the diseased pigs of our report could be related to the fact that clinical signs could lead to stress and adrenergic responses that could produce increases in this enzyme.

This report should be considered a pilot study due to the low number of animals used, and larger studies with a higher number of pigs per group should be performed to confirm these findings, since a sample size of 66 per group would be necessary to achieve 80% statistical power. In spite of this, the calculated size effects were considered as medium to high in pairwise comparisons, indicating that there was enough statistical power for the differences seen between groups. Furthermore, ideally, specific pathogen-free (SPF) animals should have been used, since commercial farm pigs may be exposed to other pathogens or environmental factors that could confound the results of this report. The absence of specific pathogen-free (SPF) animals limits the ability to strictly control variables and may introduce biases from background infections or other farm-specific conditions. However, the approach used in this report allowed us to evaluate biomarkers under real field conditions on commercial farms. Another limitation is that although this study relates biomarker changes to clinical conditions, it does not correlate them with gold standard clinical endpoints (such as histopathological findings or treatment outcomes). This limits the clinical relevance and predictive value of the biomarkers. In this line, it would be of interest to evaluate the use of these biomarkers for monitoring disease treatment in practice. In this line, this study only provides cross-sectional data at a single time point, lacking a longitudinal follow-up of biomarker changes over the course of the disease. Longitudinal data would be of high interest for understanding the temporal dynamics of biomarker responses and their relationship with disease progression and prognosis. Finally, it is important to point out that some of the biomarkers analyzed in this study could also change in stress situations, such as sAA and ADA [45]; however, regarding this, the animals in this study were under similar environmental and management conditions, and therefore it can be postulated that the stress associated with other conditions other than this disease would not be a major confounding factor in this report.

## 5. Conclusions

It can be concluded that under the conditions examined in this report, diseased animals infected by and with clinical signs of PRRS have differences in the values of selected analytes in saliva compared with pigs without clinical signs and diseased animals infected with PRRSV and clinical signs associated with *S. suis* infection. Although these findings should be tested with a larger number of animals, in a farm positive to PRRSV, the presence of increases in Hp and LDH in saliva could indicate the possibility of the activation of the disease, whereas additional increases in Serpin B12, aldolase, calprotectin, total protein, and/or ADA in saliva could indicate the presence of a bacterial disease that should be diagnosed and treated.

## Data Availability

The data presented in this study are available on request from the corresponding author.

## Abbreviations

The following abbreviations are used in this manuscript:

ADA	Adenosine deaminase
Calp	Calprotectin
CRP	C-reactive protein
Ct	Cycle threshold
*E. coli*	*Escherichia coli*
Hp	Haptoglobin
IQR	Interquartile range
LCR	Low-Complexity Region
LDH	Lactate dehydrogenase
PCR	Polymerase chain reaction
PRRS	Porcine reproductive and respiratory syndrome
PRRSV	Porcine reproductive and respiratory syndrome virus
RNA	Ribonucleic acid
RT-PCR	Real-time polymerase chain reaction
sAA	Alpha-amylase
*S. suis*	*Streptococcus suis*

## References

[1] Dee S., Brands L., Edler R., Schelkopf A., Nerem J., Spronk G., Kikuti M., Corzo C.A. (2024). Further Evidence That Science-Based Biosecurity Provides Sustainable Prevention of Porcine Reproductive and Respiratory Syndrome Virus Infection and Improved Productivity in Swine Breeding Herds. Animals.

[2] Ruedas-Torres I., Sánchez-Carvajal J.M., Salguero F.J., Pallarés F.J., Carrasco L., Mateu E., Gómez-Laguna J., Rodríguez-Gómez I.M. (2024). The Scene of Lung Pathology during PRRSV-1 Infection. Front. Vet. Sci..

[3] Meléndez A., Tejedor M.T., Mitjana O., Falceto M.V., Garza-Moreno L. (2024). Perception about the Major Health Challenges in Different Swine Production Stages in Spain. Vet. Sci..

[4] Biernacka K., Podgórska K., Tyszka A., Stadejek T. (2018). Comparison of Six Commercial ELISAs for the Detection of Antibodies against Porcine Reproductive and Respiratory Syndrome Virus (PRRSV) in Field Serum Samples. Res. Vet. Sci..

[5] Chen X., Yu Z., Li W. (2024). Molecular Mechanism of Autophagy in Porcine Reproductive and Respiratory Syndrome Virus Infection. Front. Cell. Infect. Microbiol..

[6] Sagrera M., Garza-Moreno L., Sibila M., Oliver-Ferrando S., Cárceles S., Casanovas C., Prieto P., García-Flores A., Espigares D., Segalés J. (2024). Frequency of PCV-2 Viremia in Nursery Piglets from a Spanish Swine Integration System in 2020 and 2022 Considering PRRSV Infection Status. Porc. Health Manag..

[7] Valdes-Donoso P., Jarvis L.S. (2022). Combining Epidemiology and Economics to Assess Control of a Viral Endemic Animal Disease: Porcine Reproductive and Respiratory Syndrome (PRRS). PLoS ONE.

[8] Fiers J., Cay A.B., Maes D., Tignon M. (2024). A Comprehensive Review on Porcine Reproductive and Respiratory Syndrome Virus with Emphasis on Immunity. Vaccines.

[9] Hill H., Reddick D., Caspe G., Ramage C., Frew D., Rocchi M.S., Opriessnig T., McNeilly T.N. (2024). Enhancing the Understanding of Coinfection Outcomes: Impact of Natural Atypical Porcine Pestivirus Infection on Porcine Reproductive and Respiratory Syndrome in Pigs. Virus Res..

[10] Domingo-Carreño I., Serena M.S., Martín-Valls G.E., Clilverd H., Aguirre L., Cortey M., Mateu E. (2024). The Introduction of a Highly Virulent PRRSV Strain in Pig Farms Is Associated with a Change in the Pattern of Influenza A Virus Infection in Nurseries. Vet. Res..

[11] Lebret A., Normand V., Berton P., Nicolazo T., Teixeira Costa C., Chevance C., Brissonnier M., Boulbria G. (2023). Alternative Samples for Porcine Reproductive and Respiratory Syndrome Surveillance in an Endemic PRRSV-1-Infected Breeding Herd: A Descriptive Study. Vet. Sci..

[12] Cerón J.J., Contreras-Aguilar M.D., Escribano D., Martínez-Miró S., López-Martínez M.J., Ortín-Bustillo A., Franco-Martínez L., Rubio C.P., Muñoz-Prieto A., Tvarijonaviciute A. (2022). Basics for the Potential Use of Saliva to Evaluate Stress, Inflammation, Immune System, and Redox Homeostasis in Pigs. BMC Vet. Res..

[13] López-Martínez M.J., Ornelas M.A.S., Amarie R.E., Manzanilla E.G., Martínez-Subiela S., Tecles F., Tvarijonaviciute A., Escribano D., González-Bulnes A., Cerón J.J. (2023). Changes in Salivary Biomarkers of Stress, Inflammation, Redox Status, and Muscle Damage Due to Streptococcus Suis Infection in Pigs. BMC Vet. Res..

[14] Ortín-Bustillo A., Botía M., López-Martínez M.J., Martínez-Subiela S., Cerón J.J., González-Bulnes A., Manzanilla E.G., Goyena E., Tecles F., Muñoz-Prieto A. (2023). Changes in S100A8/A9 and S100A12 and Their Comparison with Other Analytes in the Saliva of Pigs with Diarrhea Due to *E. coli*. Animals.

[15] López-Martínez M.J., Escribano D., Ortín-Bustillo A., Franco-Martínez L., González-Arostegui L.G., Cerón J.J., Rubio C.P. (2022). Changes in Biomarkers of Redox Status in Saliva of Pigs after an Experimental Sepsis Induction. Antioxidants.

[16] Gutiérrez A.M., Martínez-Subiela S., Soler L., Pallarés F.J., Cerón J.J. (2009). Use of Saliva for Haptoglobin and C-Reactive Protein Quantifications in Porcine Respiratory and Reproductive Syndrome Affected Pigs in Field Conditions. Vet. Immunol. Immunopathol..

[17] Ortín-Bustillo A., Contreras-Aguilar M.D., Rubio C.P., Botia M., Cerón J.J., López-Arjona M., Martínez-Subiela S., Escribano D., Tecles F. (2022). Evaluation of the Effect of Sampling Time on Biomarkers of Stress, Immune System, Redox Status and Other Biochemistry Analytes in Saliva of Finishing Pigs. Animals.

[18] Contreras-Aguilar M.D., Escribano D., Martínez-Subiela S., Martín-Cuervo M., Lamy E., Tecles F., Cerón J.J. (2019). Changes in Saliva Analytes in Equine Acute Abdominal Disease: A Sialochemistry Approach. BMC Vet. Res..

[19] Tecles F., Rubio C.P., Contreras-Aguilar M.D., López-Arjona M., Martínez-Miró S., Martínez-Subiela S., Cerón J.J. (2018). Adenosine Deaminase Activity in Pig Saliva: Analytical Validation of Two Spectrophotometric Assays. J. Vet. Diagn. Investig..

[20] Escribano D., Horvatić A., Contreras-Aguilar M.D., Guillemin N., Cerón J.J., Tecles F., Martinez-Miró S., Eckersall P.D., Manteca X., Mrljak V. (2019). Changes in Saliva Proteins in Two Conditions of Compromised Welfare in Pigs: An Experimental Induced Stress by Nose Snaring and Lameness. Res. Vet. Sci..

[21] Fuentes M., Tecles F., Gutiérrez A., Otal J., Martínez-Subiela S., Cerón J.J. (2011). Validation of an Automated Method for Salivary Alpha-Amylase Measurements in Pigs (*Sus scrofa domesticus*) and Its Application as a Stress Biomarker. J. Vet. Diagn. Investig..

[22] López-Martínez M.J., Cerón J.J., Ortín-Bustillo A., Escribano D., Kuleš J., Beletić A., Rubić I., González-Sánchez J.C., Mrljak V., Martínez-Subiela S. (2022). A Proteomic Approach to Elucidate the Changes in Saliva and Serum Proteins of Pigs with Septic and Non-Septic Inflammation. Int. J. Mol. Sci..

[23] Llamas-Amor E., Subiela S.M., Ramis G., Fuentes P., Goyena E., Gonzalez-Bulnes A., Manzanilla E.G., Cerón J.J., Muñoz-Prieto A., López-Martínez M.J. (2025). Serpin B12 in Saliva of Pigs: Development and Analytical Validation of an Assay for Its Quantification and Changes in SEPSIS and Stress Conditions. Res. Vet. Sci..

[24] Revisión de La Necesidad de Informar Las Estimaciones Del Tamaño Del Efecto Resumen de Algunas Medidas Recomendadas Para El Tamaño Del Efecto. https://www.researchgate.net/publication/303919832_The_need_to_report_effect_size_estimates_revisited_An_overview_of_some_recommended_measures_of_effect_size.

[25] Decorte I., Van der Stede Y., Nauwynck H., De Regge N., Cay A.B. (2013). Effect of Saliva Stabilisers on Detection of Porcine Reproductive and Respiratory Syndrome Virus in Oral Fluid by Quantitative Reverse Transcriptase Real-Time PCR. Vet. J..

[26] Almeida M.N., Zhang M., Zimmerman J.J., Holtkamp D.J., Linhares D.C.L. (2021). Finding PRRSV in Sow Herds: Family Oral Fluids vs. Serum Samples from Due-to-Wean Pigs. Prev. Vet. Med..

[27] Ortín-Bustillo A., Escribano D., López-Arjona M., Botia M., Fuentes P., Martínez-Miró S., Rubio C.P., García-Manzanilla E., Franco-Martínez L., Pardo-Marín L. (2022). Changes in a Comprehensive Profile of Saliva Analytes in Fattening Pigs during a Complete Productive Cycle: A Longitudinal Study. Animals.

[28] Steinhauser C., Yakac A.E., Markgraf W., Kromnik S., Döcke A., Talhofer P., Thiele C., Malberg H., Füssel S., Thomas C. (2024). Assessment of Hemodynamic and Blood Parameters That May Reflect Macroscopic Quality of Porcine Kidneys during Normothermic Machine Perfusion Using Whole Blood. World J. Urol..

[29] Parra M.D., Fuentes P., Tecles F., Martínez-Subiela S., Martínez J.S., Muñoz A., Cerón J.J. (2006). Porcine Acute Phase Protein Concentrations in Different Diseases in Field Conditions. J. Vet. Med. Ser. B.

[30] Tor M., Fraile L., Vilaró F., Pena R.N. (2024). Multiplex Assay to Determine Acute Phase Proteins in Modified Live PRRSV Vaccinated Pigs. J. Proteome Res..

[31] Saco Y., Martínez-Lobo F., Cortey M., Pato R., Peña R., Segalés J., Prieto C., Bassols A. (2016). C-Reactive Protein, Haptoglobin and Pig-Major Acute Phase Protein Profiles of Pigs Infected Experimentally by Different Isolates of Porcine Reproductive and Respiratory Syndrome Virus. Vet. Microbiol..

[32] Zheng H.-Z., Miao X., Chang J., Zhou H., Zhang J.-J., Mo H.-M., Jia Q. (2024). Smoking Behavior Associated Upregulation of SERPINB12 Promotes Proliferation and Metastasis via Activating WNT Signaling in NSCLC. J. Cardiothorac. Surg..

[33] Gong X., Liang Y., Wang J., Pang Y., Wang F., Chen X., Zhang Q., Song C., Wang Y., Zhang C. (2024). Highly Pathogenic PRRSV Upregulates IL-13 Production through Nonstructural Protein 9–Mediated Inhibition of N6-Methyladenosine Demethylase FTO. J. Biol. Chem..

[34] Asai T., Mori M., Okada M., Uruno K., Yazawa S., Shibata I. (1999). Elevated Serum Haptoglobin in Pigs Infected with Porcine Reproductive and Respiratory Syndrome Virus. Vet. Immunol. Immunopathol..

[35] Díaz I., Darwich L., Pappaterra G., Pujols J., Mateu E. (2005). Immune Responses of Pigs after Experimental Infection with a European Strain of Porcine Reproductive and Respiratory Syndrome Virus. J. Gen. Virol..

[36] Pirovich D.B., Da’dara A.A., Skelly P.J. (2021). Multifunctional Fructose 1,6-Bisphosphate Aldolase as a Therapeutic Target. Front. Mol. Biosci..

[37] Song Z., Bai J., Liu X., Nauwynck H., Wu J., Liu X., Jiang P. (2019). S100A9 Regulates Porcine Reproductive and Respiratory Syndrome Virus Replication by Interacting with the Viral Nucleocapsid Protein. Vet. Microbiol..

[38] Lamot M., Miler M., Nikolac Gabaj N., Lamot L., Milošević M., Harjaček M., Abdović S. (2022). Serum Calprotectin Is a Valid Biomarker in Distinction of Bacterial Urinary Tract Infection from Viral Respiratory Illness in Children Under 3 Years of Age. Front. Pediatr..

[39] Sali V., Veit C., Valros A., Junnikkala S., Heinonen M., Nordgreen J. (2021). Dynamics of Salivary Adenosine Deaminase, Haptoglobin, and Cortisol in Lipopolysaccharide-Challenged Growing Pigs. Front. Vet. Sci..

[40] Bradford K.L., Moretti F.A., Carbonaro-Sarracino D.A., Gaspar H.B., Kohn D.B. (2017). Adenosine Deaminase (ADA)-Deficient Severe Combined Immune Deficiency (SCID): Molecular Pathogenesis and Clinical Manifestations. J. Clin. Immunol..

[41] Liu Q., Yu Y.-Y., Wang H.-Y. (2022). Expression of the Highly Pathogenic Porcine Reproductive and Respiratory Syndrome Virus (HP-PRRSV) in Various Types of Cells in Thymic Tissues. Pol. J. Vet. Sci..

[42] Hovi T., Smyth J.F., Allison A.C., Williams S.C. (1976). Role of Adenosine Deaminase in Lymphocyte Proliferation. Clin. Exp. Immunol..

[43] Liu J., Su G., Duan C., Sun Z., Xiao S., Zhou Y., Fang L. (2024). Porcine Reproductive and Respiratory Syndrome Virus Infection Activates ADAM17 to Induce Inflammatory Responses. Vet. Microbiol..

[44] Contreras-Aguilar M.D., Tvarijonaviciute A., Monkeviciene I., Martín-Cuervo M., González-Arostegui L.G., Franco-Martínez L., Cerón J.J., Tecles F., Escribano D. (2020). Characterization of Total Adenosine Deaminase Activity (ADA) and Its Isoenzymes in Saliva and Serum in Health and Inflammatory Conditions in Four Different Species: An Analytical and Clinical Validation Pilot Study. BMC Vet. Res..

[45] Contreras-Aguilar M.D., Escribano D., Martínez-Miró S., López-Arjona M., Rubio C.P., Martínez-Subiela S., Cerón J.J., Tecles F. (2019). Application of a Score for Evaluation of Pain, Distress and Discomfort in Pigs with Lameness and Prolapses: Correlation with Saliva Biomarkers and Severity of the Disease. Res. Vet. Sci..

[46] Jurkovich V., Hejel P., Kovács L. (2024). A Review of the Effects of Stress on Dairy Cattle Behaviour. Animals.

